# Impact of chronic obstructive pulmonary disease in patients with atrial fibrillation: an analysis from the GLORIA-AF registry

**DOI:** 10.1093/europace/euae021

**Published:** 2024-01-24

**Authors:** Giulio Francesco Romiti, Bernadette Corica, Davide Antonio Mei, Frederick Frost, Arnaud Bisson, Giuseppe Boriani, Tommaso Bucci, Brian Olshansky, Tze-Fan Chao, Menno V Huisman, Marco Proietti, Gregory Y H Lip

**Affiliations:** Liverpool Centre for Cardiovascular Science at University of Liverpool, Liverpool John Moores University and Liverpool Heart & Chest Hospital, Liverpool, UK; Department of Translational and Precision Medicine, Sapienza – University of Rome, Rome, Italy; Liverpool Centre for Cardiovascular Science at University of Liverpool, Liverpool John Moores University and Liverpool Heart & Chest Hospital, Liverpool, UK; Department of Translational and Precision Medicine, Sapienza – University of Rome, Rome, Italy; Department of Translational and Precision Medicine, Sapienza – University of Rome, Rome, Italy; Cardiology Division, Department of Biomedical, Metabolic and Neural Sciences, University of Modena and Reggio Emilia, Policlinico di Modena, Modena, Italy; Liverpool Centre for Cardiovascular Science at University of Liverpool, Liverpool John Moores University and Liverpool Heart & Chest Hospital, Liverpool, UK; Liverpool Centre for Cardiovascular Science at University of Liverpool, Liverpool John Moores University and Liverpool Heart & Chest Hospital, Liverpool, UK; Service de Cardiologie, Centre Hospitalier Régional Universitaire et Faculté de Médecine de Tours, Tours, France; Cardiology Division, Department of Biomedical, Metabolic and Neural Sciences, University of Modena and Reggio Emilia, Policlinico di Modena, Modena, Italy; Liverpool Centre for Cardiovascular Science at University of Liverpool, Liverpool John Moores University and Liverpool Heart & Chest Hospital, Liverpool, UK; Department of General Surgery and Surgical Specialties ‘Paride Stefanini’, Sapienza – University of Rome, Rome, Italy; Division of Cardiology, Department of Medicine, University of Iowa, Iowa City, USA; Division of Cardiology, Department of Medicine, Taipei Veterans General Hospital, Taipei, Taiwan; Institute of Clinical Medicine, and Cardiovascular Research Center, National Yang Ming Chiao Tung University, Taipei, Taiwan; Department of Thrombosis and Hemostasis, Leiden University Medical Center, Leiden, The Netherlands; Department of Clinical Sciences and Community Health, University of Milan, Milan, Italy; Division of Subacute Care, IRCCS Istituti Clinici Scientifici Maugeri, Milan, Italy; Liverpool Centre for Cardiovascular Science at University of Liverpool, Liverpool John Moores University and Liverpool Heart & Chest Hospital, Liverpool, UK; Danish Center for Health Services Research, Department of Clinical Medicine, Aalborg University, Aalborg, Denmark

**Keywords:** Atrial fibrillation, Chronic obstructive pulmonary disease, COPD, Oral anticoagulant, Prognosis

## Abstract

**Aims:**

Chronic obstructive pulmonary disease (COPD) may influence management and prognosis of atrial fibrillation (AF), but this relationship has been scarcely explored in contemporary global cohorts. We aimed to investigate the association between AF and COPD, in relation to treatment patterns and major outcomes.

**Methods and results:**

From the prospective, global GLORIA-AF registry, we analysed factors associated with COPD diagnosis, as well as treatment patterns and risk of major outcomes in relation to COPD. The primary outcome was the composite of all-cause death and major adverse cardiovascular events (MACEs). A total of 36 263 patients (mean age 70.1 ± 10.5 years, 45.2% females) were included; 2,261 (6.2%) had COPD. The prevalence of COPD was lower in Asia and higher in North America. Age, female sex, smoking, body mass index, and cardiovascular comorbidities were associated with the presence of COPD. Chronic obstructive pulmonary disease was associated with higher use of oral anticoagulant (OAC) [adjusted odds ratio (aOR) and 95% confidence interval (CI): 1.29 (1.13–1.47)] and higher OAC discontinuation [adjusted hazard ratio (aHR) and 95% CI: 1.12 (1.01–1.25)]. Chronic obstructive pulmonary disease was associated with less use of beta-blocker [aOR (95% CI): 0.79 (0.72–0.87)], amiodarone and propafenone, and higher use of digoxin and verapamil/diltiazem. Patients with COPD had a higher hazard of primary composite outcome [aHR (95% CI): 1.78 (1.58–2.00)]; no interaction was observed regarding beta-blocker use. Chronic obstructive pulmonary disease was also associated with all-cause death [aHR (95% CI): 2.01 (1.77–2.28)], MACEs [aHR (95% CI): 1.41 (1.18–1.68)], and major bleeding [aHR (95% CI): 1.48 (1.16–1.88)].

**Conclusion:**

In AF patients, COPD was associated with differences in OAC treatment and use of other drugs; Patients with AF and COPD had worse outcomes, including higher mortality, MACE, and major bleeding.

## Graphical Abstract

**Graphical Abstract:**
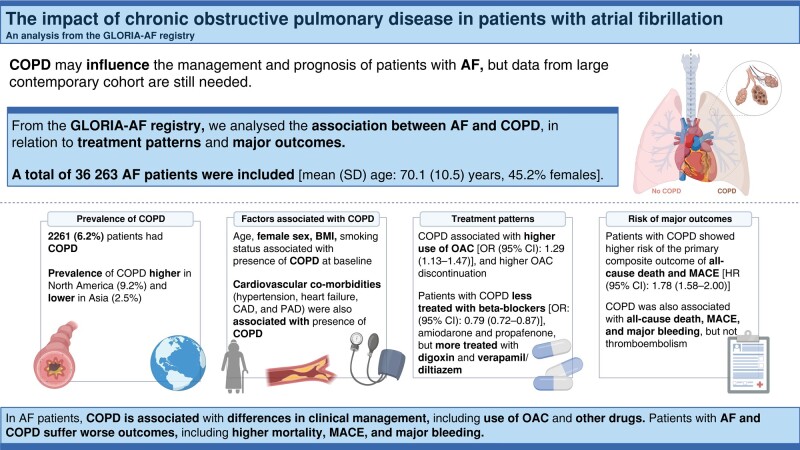
AF = Atrial Fibrillation; CAD = Coronary Artery Disease; CI = Confidence Interval; COPD = Chronic Obstructive Pulmonary Disease; HR = Hazard Ratio; MACE = Major Adverse Cardiovascular Events; OAC = Oral Anticoagulant; OR = Odds Ratio; PAD = Peripheral Artery Disease; SD, standard deviation. (created with Biorender.com).

**What’s new?**

- The relationship between chronic obstructive pulmonary disease (COPD) and atrial fibrillation (AF) is not completely understood.
- In patients with AF, COPD is common and influences clinical management.
- Patients with AF and COPD showed higher risk of death, cardiovascular events, and major bleeding.
- The relationship between AF and COPD requires specific attention to improve prognosis.

## Introduction

The relationship between atrial fibrillation (AF) and chronic obstructive pulmonary disease (COPD) has gained increasing attention in recent years.^1^ In patients with AF, COPD is one of the most common non-cardiovascular comorbidities, and, whilst epidemiological studies report a global prevalence of COPD in patients with AF of 13%,^2^ substantial geographical variation has been described, with higher prevalence in Western countries,^2–4^ reflecting the global epidemiological trends of COPD.^4^ These numbers, along with the progressive ageing of the population and the increasing incidence of AF,^5^ set the stage for an emerging ‘AF–COPD syndemic’.

Over the last decades, evidence of the detrimental effect of COPD on management and outcomes of patients with AF has bolstered interest in the relationship between AF and COPD.^2^ Indeed, COPD has been linked to challenging management issues and suboptimal treatment in patients with AF, as well as differences in drug prescriptions, including beta-blockers and beta-2 agonists, based on concerns about safety.^1,6,7^ Several mechanisms may explain the deleterious interaction between COPD and AF, including a role of inflammation that may promote atrial remodelling and fibrosis, and the contribution of autonomic dysfunction, hypoxia, and intra-thoracic pressure abnormalities.^1,8–10^ Consistently, COPD has been repeatedly considered a key predictor of incident AF and progression to its more sustained forms,^11,12^ poorer outcomes and more recurrences after AF ablation,^13^ and an overall higher risk of adverse consequences, including death.^2^

Notwithstanding previous evidence, there remains uncertainty regarding the epidemiology of the AF–COPD association. Data from large, contemporary, and global cohorts may improve our understanding of this relationship and clarify the unmet management needs of patients with AF and COPD.

In this study, we analysed associations between COPD and AF data from the *Global Registry on Long-Term Antithrombotic Treatment in Patients with Atrial Fibrillation* (GLORIA-AF) Phase II and Phase III registry to assess the management and prognosis of these patients.

## Methods

The GLORIA-AF registry, an international, prospective, multi-centre registry programme structured in three phases, aimed to evaluate real-world long-term efficacy and safety of dabigatran etexilate in patients with AF. Details on the design, follow-up, and primary results of GLORIA-AF registry were previously published.^14–17^ During the study periods (2011–14 for Phase II and 2014–16 for Phase III), adults (≥18 years) with a recent diagnosis of non-valvular AF (i.e. within 3 months or 4.5 months in Latin America) and a CHA_2_DS_2_-VASc score of ≥1 were consecutively enrolled. The main exclusion criteria were AF due to a reversible cause, mechanical heart valve (or patients expected to undergo valve replacement), prior treatment with VKA for >60 days during lifetime, other clinical indication for oral anticoagulant (OAC) treatment, or short life expectancy (<1 year). The study protocol was approved by local institutional review boards at each participating centre, and the study was conducted according to the Good Clinical Practice and the Declaration of Helsinki. All patients provided written informed consent.

### Chronic obstructive pulmonary disease and treatments

At baseline, investigators recorded data regarding demographics, comorbidities, and treatment prescribed for patients enrolled in the study, using standardized electronic case report forms. Amongst co-morbidities, investigators were able to report whether patients had respiratory diseases and specifically if patients had COPD; no additional information on the definition or severity of COPD was available. Smoking status [either non-smoker (<100 cigarettes in lifetime), current smoker, past smoker, or unknown] was also collected. As per treatments, in this analysis, we considered antithrombotic use, as well as concomitant treatment with cardiovascular drugs [i.e. angiotensin-converting enzyme (ACE) inhibitors, angiotensin receptor blockers, diuretics, beta-blockers [either selective or non-selective], digoxin, verapamil/diltiazem, propafenone, flecainide, amiodarone, dronedarone, and other antiarrhythmics). We additionally analysed data regarding the use of ablation/cardioversion in these patients.

### Follow-up and outcomes

Antithrombotic treatment discontinuation and major clinical outcomes were recorded during follow-up. During Phase II of the GLORIA-AF registry, a 2-year follow-up was performed only for patients prescribed dabigatran at baseline. During Phase III, all patients (regardless of antithrombotic therapy received) were followed up for 3 years.

In this analysis, we analysed treatment discontinuation at 24 months only for those patients who received OAC at baseline. Consistent with previous analyses,^18^ we defined discontinuation as switching to another antithrombotic regimen (including different OAC), or an interruption longer than 30 days of the treatment received at baseline.

We defined our *primary outcome* as the composite of all-cause death, stroke, and myocardial infarction. We also explored secondary outcomes: (i) all-cause mortality, (ii) major adverse cardiovascular events (MACEs, defined as the composite of cardiovascular death, stroke, and myocardial infarction), (iii) thromboembolism [i.e. a composite of stroke, transient ischaemic attack (TIA), and other non-central nervous system thromboembolism], and (iv) major bleeding (defined as a life-threatening or fatal bleeding, symptomatic bleeding in a critical organ, or bleeding associated with a haemoglobin reduction of ≥20 g/L or leading to ≥2 unit of blood transfusion).

### Statistical analysis

Continuous variables were reported as mean and standard deviation and compared using a parametric test or median and interquartile range (IQR) and compared with non-parametric test, if non-normally distributed. Binary and categorical variables were reported as frequencies and percentages and were compared using a chi-square test.

We evaluated factors associated with the presence of COPD at baseline using a multiple logistic regression model. Covariates included in the model were the components of the of CHA_2_DS_2_-VASc score [age <65, 65–75, or ≥75 years, sex, arterial hypertension, diabetes, heart failure, coronary artery disease (CAD), history of stroke/TIA, and peripheral artery disease (PAD)], phase of recruitment, type of AF (paroxysmal, persistent, or permanent), history of previous bleeding, geographical region of recruitment, history of smoking, and body mass index (BMI), modelled as a restricted cubic spline with four knots. Results were reported as adjusted odds ratio (aOR) and 95% confidence intervals (CIs).

Multiple logistic regression models were also used to estimate odds of treatment prescription in patients with vs. without COPD at baseline. Other covariates included were components of the CHA_2_DS_2_-VASc score, phase of recruitment, type of AF, BMI, and history of previous bleeding. Results were reported as aOR and 95% CI.

The associations between COPD and OAC discontinuation and major outcomes were evaluated using Cox regression models. Other covariates included were components of the CHA_2_DS_2_-VASc score, phase of recruitment, type of AF, BMI, and history of previous bleeding. For the models evaluating the risk of major outcomes, we also included treatment with OAC. Results were reported as adjusted hazard ratios (aHRs) and 95% CI.

For the primary outcome, we additionally reported Kaplan–Meier curves, with survival distributions compared using the log-rank test. Moreover, we also explored the interactions between COPD, relevant baseline characteristics (i.e. age, sex, geographical location, race/ethnicity, CHA_2_DS_2_-VASc score, type of AF, CAD, heart failure, phase of recruitment, treatment with OAC, and beta-blocker), and the risk of the primary composite outcome.

A two-sided *P* < 0.05 was considered statistically significant. All the analyses were performed using R 4.3.1 (R Core Team 2020, Vienna, Austria).

## Results

A total of 36 263 patients (mean age 70.1 ± 10.5 years, 45.2% females) who were enrolled in the GLORIA-AF registry Phase II and Phase III and had available data on the presence of COPD at baseline were included in this analysis; 2261 (6.2%) were reported to have COPD.

Supplementary material online, *Table S1* shows baseline characteristics according to the presence of COPD. The prevalence of COPD was reported to be highest in patients recruited in North America (9.2%) and lowest in those recruited in Asia (2.5%). Male sex was more represented in patients with COPD (60.8% vs. 54.3% in patients with vs. without COPD, *P* < 0.001). Patients with COPD had a more frequent history of current or past smoking and higher prevalence of most co-morbidities, including hypertension, heart failure, CAD, and diabetes mellitus. Consistently, a higher proportion of patients with COPD had a CHA_2_DS_2_-VASc score of ≥2 (92.8% vs. 85.3% in patients without COPD, *P* < 0.001).

### Factors associated with the presence of chronic obstructive pulmonary disease

**Figure 1:**
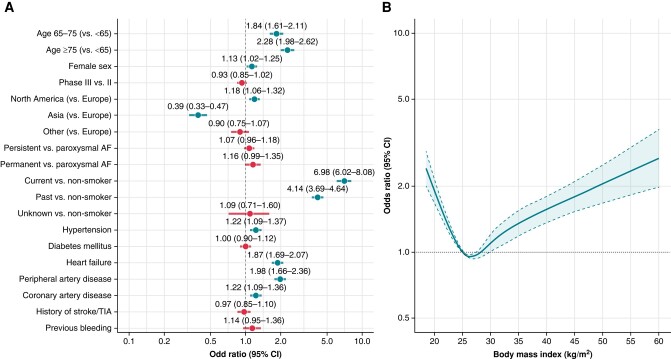
Factors associated with the presence of chronic obstructive pulmonary disease at baseline. (*A*) Categorical variables. (*B*) Body mass index. AF, atrial fibrillation; CI, confidence interval; TIA, transient ischaemic attack.

In a multivariable logistic regression model (*Figure 1*), we found that the presence of COPD at baseline was associated with increasing age [aOR (95% CI) 1.84 (1.61–2.11) and 2.28 (1.98–2.62) for 65–75 vs. <65 years and ≥75 vs. 65 years, respectively], female sex, and history of current and past smoking. Recruitment in North America was associated with higher odds of COPD diagnosis [aOR (95% CI): 1.18 (1.06–1.32)]; conversely, recruitment in Asia was inversely associated with COPD at baseline [aOR (95% CI): 0.39 (0.33–0.47)]. Amongst co-morbidities, hypertension, heart failure, CAD, and PAD were found associated with COPD diagnosis. We observed a J-shaped, non-linear relationship between BMI and the odds of COPD (*Figure 1B*).

### Association of chronic obstructive pulmonary disease with pharmacological treatments and management

**Figure 2:**
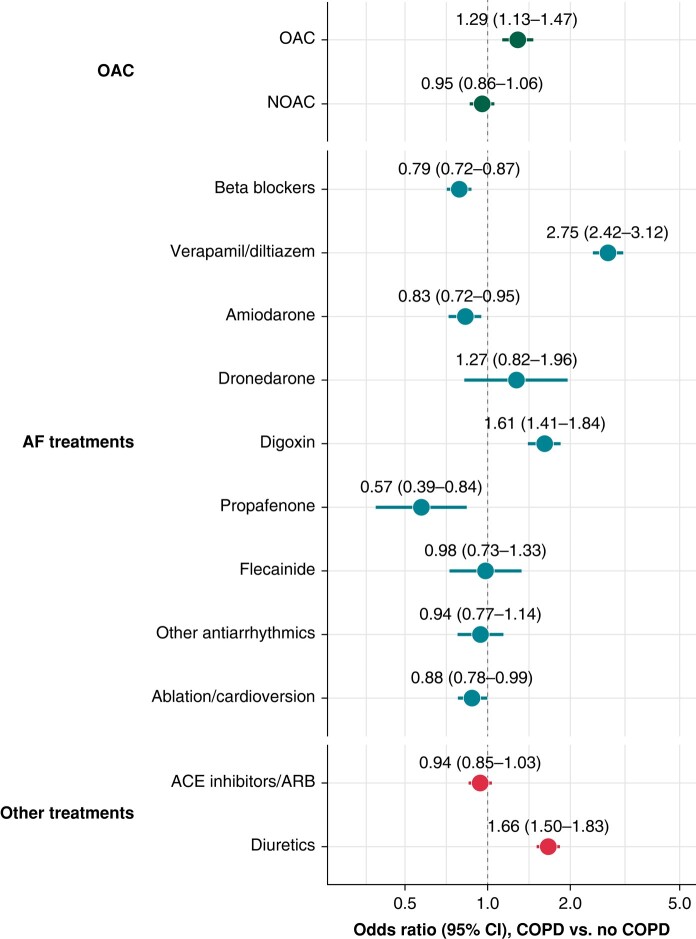
Association between chronic obstructive pulmonary disease and treatment received at baseline. ACE, angiotensin-converting enzyme; AF, atrial fibrillation; ARB, angiotensin receptor blockers; CI, confidence interval; COPD, chronic obstructive pulmonary disease; NOAC, non-vitamin K antagonist oral anticoagulant; OAC, oral anticoagulant.

Treatments according to the diagnosis of COPD are reported in Supplementary material online, *Table S2*, whilst results of the multiple logistic regression model are reported in *Figure 2*. Chronic obstructive pulmonary disease was associated with higher OAC use [aOR (95% CI): 1.29 (1.13–1.47)], with no significant differences observed for the type of OAC used.

At 24 months, rates of OAC discontinuation were higher in patients with COPD (28.9% vs. 27.2%, Supplementary material online, *Figure S1*); on multivariable Cox regression analysis, we found that COPD was associated with higher hazard of OAC discontinuation during follow-up [aHR (95% CI): 1.12 (1.01–1.25)].

Chronic obstructive pulmonary disease was associated with lower odds of treatment with beta-blockers [aOR: 0.79 (0.72–0.87), *Figure 2*], as well as amiodarone and propafenone; we also observed marginally statistically significant lower odds of having received AF ablation or electrical cardioversion [aOR (95% CI): 0.88 (0.78–0.99)]. Conversely, COPD was associated with higher odds of receiving verapamil or diltiazem [aOR (95% CI): 2.75 (2.42–3.12)] and digoxin [aOR (95% CI): 1.61 (1.41–1.84)], as well as diuretics [aOR (95% CI): 1.66 (1.50–1.83)].

### Risk of adverse outcomes

A total of 25 860 patients (71.3%) with available follow-up data on the risk of the primary composite outcome were included in the survival analysis, with a median follow-up of 3.0 years (IQR: 2.1–3.1). Amongst patients not included in the survival analysis, a higher proportion was female and had COPD; no statistically significant differences were observed for age and mean CHA_2_DS_2_-VASc score. Patients excluded also had slightly higher prevalence of COPD, heart failure, CAD, and history of bleeding (see Supplementary material online, *Table S3*).

**Figure 3:**
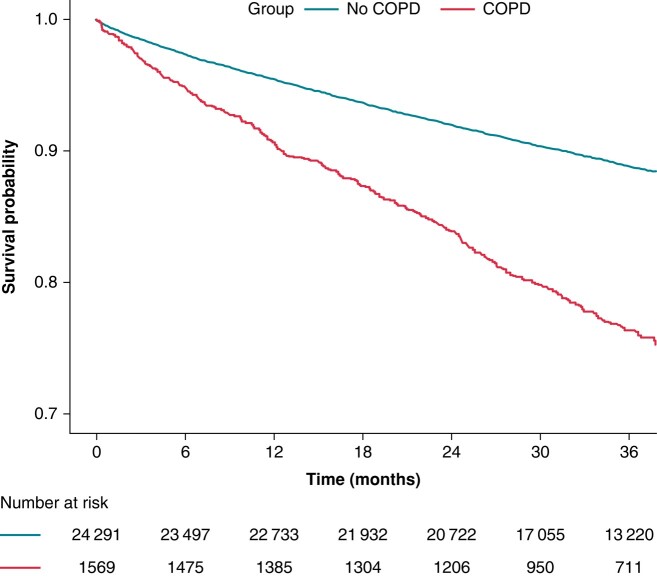
Kaplan–Meier curves for the primary composite outcome of all-cause death and major adverse cardiovascular event according to latent classes. Log-rank: 216.44, *P* < 0.001. COPD, chronic obstructive pulmonary disease.

Survival curves for the primary composite outcome are reported in *Figure 3*. Patients with COPD showed a lower survival probability during follow-up (log-rank: 216.44, *P* < 0.001). Multiple adjusted Cox regression analysis (*Table 1*) showed that COPD was associated with a higher hazard of the primary composite outcome [aHR (95% CI): 1.78 (1.58–2.00)]. Similar results were observed for the exploratory secondary outcomes, with COPD associated with increased hazard of all-cause death at adjusted Cox regression analysis [aHR (95% CI): 2.01 (1.77–2.28)], MACE [aHR (95% CI): 1.41 (1.18–1.68)], and major bleeding [aHR (95% CI): 1.48 (1.16–1.88)]; no statistically significant differences were observed for thromboembolism.

**Table 1:** Multiple Cox regressions on the risk of major outcomes according to the presence of chronic obstructive pulmonary disease

	Incidence rate per 100 persons-years (95% CI)	Adjusted hazard ratio (95% CI)^a^	*P*
**Primary outcome**			
*Composite of all-cause death, stroke, and MI*			
No COPD	4.0 (3.9–4.2)	Ref.	
COPD	9.1 (8.2–10.1)	**1.78 (1.58–2.00)**	**<0**.**001**
**Secondary outcomes**			
*All-cause death*			
No COPD	3.0 (2.8–3.1)	Ref.	
COPD	7.8 (7.0–8.8)	**2.01 (1.77–2.28)**	**<0**.**001**
*MACE*			
No COPD	2.2 (2.1–2.3)	Ref.	
COPD	3.9 (3.3–4.5)	**1.41 (1.18–1.68)**	**<0**.**001**
*Thromboembolism*			
No COPD	1.3 (1.2–1.4)	Ref.	
COPD	1.5 (1.1–1.9)	1.06 (0.80–1.40)	0.682
*Major bleeding*			
No COPD	1.2 (1.1–1.2)	Ref.	
COPD	2.2 (1.7–2.7)	**1.48 (1.16–1.88)**	**0**.**002**

^a^Adjusted for age, sex, phase of recruitment, type of AF, BMI, history of hypertension, diabetes, heart failure, coronary artery disease, peripheral artery disease, stroke/TIA and previous bleeding, and use of OAC. Bold text depicts statistically significant results at *P* < 0.05 level.

CI, confidence interval; COPD, chronic obstructive pulmonary disease; IR, incidence rate; MI, myocardial infarction; Ref, reference.

**Figure 4:**
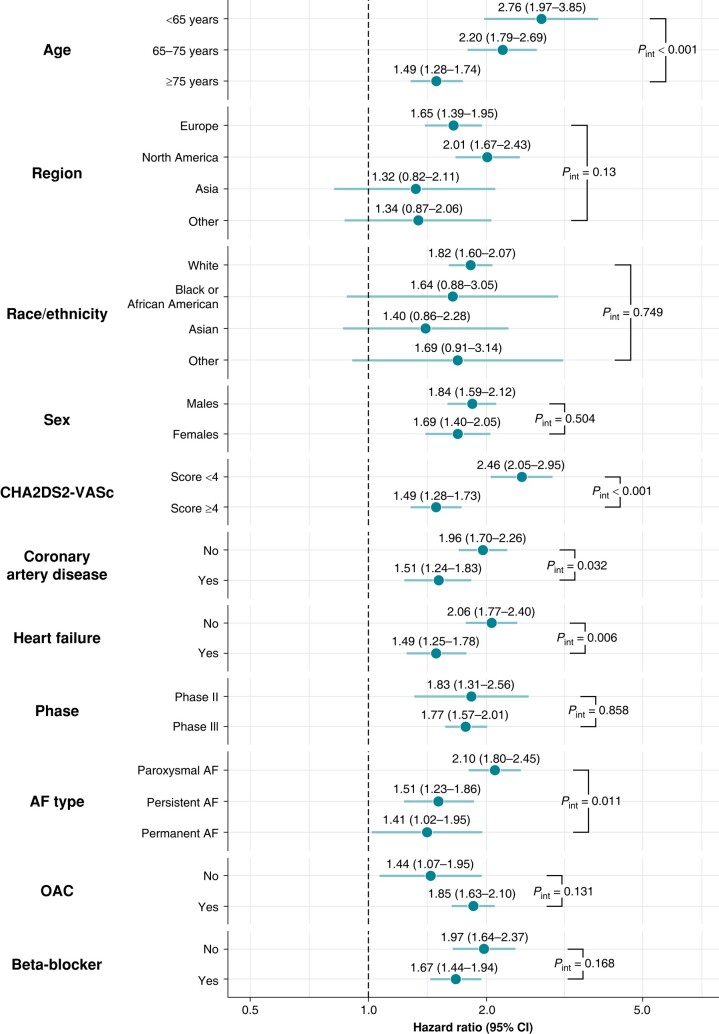
Interaction analysis on the risk of the primary outcome according to the presence of chronic obstructive pulmonary disease. AF, atrial fibrillation; OAC, oral anticoagulant.

Results of the interaction analysis between relevant characteristics, COPD, and the risk of the primary outcome are reported in *Figure 4*. In patients with a CHA_2_DS_2_-VASc score of <4, the association between COPD and risk of the primary outcomes was higher in magnitude, compared with patients with a CHA_2_DS_2_-VASc score of ≥4 (*P*_int_ < 0.001); similar results were found according to age and in patients without CAD, without heart failure, and with paroxysmal AF (*P*_int_ < 0.001, *P*_int_ = 0.032, *P*_int_ = 0.006, and *P*_int_ = 0.011, respectively). No statistically significant interactions were observed according to treatment with OAC (*P*_int_ = 0.131) or beta-blockers (*P*_int_ = 0.168), nor according to region or phase of recruitment, race/ethnicity, or sex.

## Discussion

In this analysis from a global and contemporary cohort of recently diagnosed AF patients, our main findings are as follows: (i) COPD was reported in 1 out of 16 patients with AF, with higher prevalence observed in patients enrolled in North America; (ii) at multivariable logistic regression analysis, increasing age, female sex, history of smoking, and cardiovascular comorbidities (i.e. arterial hypertension, heart failure, CAD, and PAD) were all associated with COPD, whilst BMI showed a J-shaped relationship; (iii) COPD was associated with higher use of OAC as well as marginally significant higher OAC discontinuation and an overall differential drug management of AF; (iv) patients reported as having COPD had a worse prognosis at multiple-adjusted Cox regression, including higher risk of all-cause death, MACE, and major bleeding.

Previous estimates of the prevalence of COPD in patients with AF were as high as 13%, with significant heterogeneity, largely driven by geographical setting.^2^ In our study, we found a lower prevalence of COPD (6.2%), with higher figures in North America and lower in Asia.^2^ Our prevalence appears similar to other observational registries, such as the EORP-AF Long-Term^19^ and the Japanese Fushimi-AF^20^ registries, suggesting that detection and reporting of COPD in observational studies may partly explain the relatively low prevalence found. Nonetheless, misdiagnosis of COPD in general care and primary setting has been previously reported.^21,22^

We found that several clinical factors were associated with COPD in patients with AF. Whilst age and history of smoking are amongst the most common risk factors for COPD,^4^ we also observed higher odds of COPD in females at multivariable analysis. This result seems consistent with the mean age of our population and with recent epidemiological data showing how COPD is more common in women amongst elderly patients.^4^ Finally, various cardiovascular conditions were all independently associated with COPD at baseline. Overall, these findings suggest that COPD is more common in patients with higher morbidity: indeed, comorbidities in patients with AF do not occur in isolation but tend to ‘cluster’, ultimately entailing the so-called clinical complexity that has a detrimental effect on the management and prognosis of AF patients.^23–26^ Of note, both AF and COPD are associated with multi-morbidity, a common theme linking these two diseases.^3,27,28^

We also found relevant differences in the management and outcomes according to the presence of COPD. In our cohort, patients with COPD had higher use of OACs, as well as less odds of receiving beta-blockers, in accordance with previous studies and meta-analysis.^2,29,30^ Nonetheless, prescription patterns may be influenced by local practices, with other studies showing lower use of OACs in patients with COPD and AF.^31^ Finally, we observed differences in the use of other treatments, including drugs used for rhythm control, and ablation/cardioversion procedures. The lower use of treatments and interventions for rhythm control, such as antiarrhythmic drugs, cardioversion, and AF ablation, could have contributed to the worse outcome observed in COPD patients, considering their effect on prognosis in patients with AF.^32–34^

Taken together, these findings suggest that the profile and characteristics of COPD patients may influence physicians’ decisions on AF management. Indeed, some hypotheses can explain our results. Some reports have previously associated COPD with a higher risk of AF recurrence after ablation^13,35,36^ and lower rates of successful electrical cardioversion,^37^ although sometimes with conflicting results.^38,39^ Concerns on the efficacy of such procedures in patients with COPD may therefore explain the lower use that we observed in these subjects. Similarly, several hypotheses can explain the lower odds of receiving some drugs that we observed in patients with COPD. For instance, amiodarone has known lung side-effects,^40^ and caution has also been advocated when using propafenone in patients with COPD.^41^ Although we cannot draw definitive conclusions on reasons underlying the lower use of these drugs that we observed in patients with COPD, such factors may have played a role. On the other side, the lower use of beta-blockers in patients with COPD has been already reported,^42^ and the contemporary higher use of other drugs, such as digoxin and verapamil/diltiazem, may reflect different choices of physicians in this scenario. Finally, the association between COPD and higher use of diuretics was previously reported^43^ and may reflect the complex interplay between AF, impaired cardiac function (as in heart failure), and respiratory symptoms, underlining the challenging management of these patients.

These results have clinical significance. Whilst available evidence suggests that beta-blockers (particularly, beta-1 selective ones) are safe and even associated with reduced mortality in patients with COPD and cardiovascular disease,^2,44,45^ under-prescription has been previously described,^2^ even in a recent Danish nationwide analysis of patients with COPD.^30^ Our results confirm and expand these findings, suggesting that COPD may complicate patient management by influencing treatment choices, thus contributing to the complexity of patients with AF and COPD.

The association between COPD and worse prognosis has also important clinical implications. Patients with COPD had a higher hazard of all-cause death, MACEs, and major bleeding, but not thromboembolism. These findings, and the magnitude of the associations, are in line with a previous comprehensive meta-analysis^2^ as well as other studies^46–48^ and confirm that COPD has a detrimental effect in patients with AF. This association can be explained by a direct effect of COPD on prognosis^1,49^ and by the overall complexity and dynamic interplay between AF and COPD. Indeed, AF itself could complicate the management of COPD^1^ and has been associated with higher mortality after COPD exacerbations,^50^ suggesting that AF and COPD may exert a bidirectional detrimental effect on prognosis. Moreover, COPD has been associated with frailty, multi-morbidity, and higher use of healthcare resources, especially when exacerbations occur^51–53^; this can further complicate the natural history of patients with AF and COPD.

The association of COPD diagnosis with the risk of major outcomes was notably greater in patients with lower CHA_2_DS_2_-VASc scores and those without CAD or heart failure. This was likely due to the lower total risk of adverse events from other causes in these patients. Other factors (including treatment with OACs and beta-blockers) did not appear to significantly modify the association between COPD and prognosis in patients with AF.

Our study underlines the complexity arising by the contemporary diagnosis of COPD in patients with AF. Indeed, to address complexities in the management of AF, the ‘Atrial fibrillation Better Care’ (ABC) pathway has been proposed, to streamline holistic care encompassing optimal stroke prevention, patient-centred decision AF control strategies, and optimization of the management of other comorbidities and risk factors.^54^ The ABC pathway has been associated with better outcomes in patients with AF,^55–58^ even in those with multi-morbidity or deemed as ‘clinically complex’.^24,59–61^ The poor prognostic outcomes of people living with COPD and AF seen here suggest that COPD may be a key targetable comorbidity for optimization. A number of evidence-based interventions are available for people with COPD including pulmonary rehabilitation, smoking cessation, oxygen therapy, and lung volume reduction surgery, all of which have been shown to reduce all-cause mortality.^62–65^ More recently, the ETHOS study demonstrated triple inhaled therapy reduced all-cause mortality vs. dual bronchodilator therapy, with a reduction in cardiovascular deaths accounting for the majority of the reductions.^66,67^ These findings have led to calls for cardiovascular risk to be considered as a novel key component of COPD disease classification.^68^

Taken together, whilst we do not have data to evaluate the effect of COPD-specific treatments in our cohort, our results suggest that a greater recognition of this relationship in clinical practice, a more accurate identification of COPD, and the implementation of holistic and integrated approaches for the management of these patients—as recommended by international guidelines^69,70^—could potentially counteract the detrimental effect of COPD in patients with AF. Whilst further studies are needed to verify this hypothesis, implementation of such an approach in patients with AF and COPD appears rationale, as it may lead to a more integrated management of these patients and ultimately potential improvements in healthcare resources use and prognosis, also considering different practices in the management of AF across different specialties.^71,72^ Although evidence is needed to demonstrate the beneficial effect of such approach in patients with AF and COPD, recent attempts at implementing a screening and management pathway for COPD in patients with AF have shown promising results^73^ and particularly in improving diagnosis of respiratory conditions, including COPD.

### Strengths and limitations

Our manuscript provides a comprehensive analysis on the relationship between COPD and AF, in a large, global, and contemporary cohort of patients with AF. This contributes to strengthen the generalizability of our results, also in view of previous evidence.

Nonetheless, we acknowledge some limitations. First, this is a *post hoc* analysis of a prospective observational study, and we may have limited power to observe differences between the groups. Second, we defined COPD as per the data collected at baseline by each investigator, and we have no information on the severity or length of the disease or on respiratory functional assessments. This could have led to under-diagnosis of COPD or enrichment of our data set with more severe cases of COPD, potentially influencing our results. Overall, this highlights a systemic issue regarding limited extra-cardiac information in heart disease registries. Moreover, we had no data on the type of beta-blockers (either beta-1 selective or not) received, and neither on treatment with beta-2 agonists or other COPD-specific agents, including the use of corticosteroids (whether systemic or inhaling ones). Further studies with more granular data—particularly regarding treatments for COPD—are needed to explore the use of these drugs in patients with COPD and AF, also considering that previous data show how the detrimental association between COPD and AF may be enhanced in patients with frequent exacerbations and higher inflammation.^74^ Whilst our regression analyses were adjusted for several factors, we cannot exclude the contribution of other unaccounted confounders on the results observed. Finally, our findings were not adjusted for multiple comparisons and as such should be regarded as exploratory and interpreted with caution.

## Conclusions

In this analysis from a large, global, and contemporary registry, COPD was identified in 1 out of 16 patients with AF and was associated with differences in drug therapies, with higher OAC use. Such patients had a higher mortality and greater risk of MACE and major bleeding.

## Supplementary material

Supplementary material is available at *Europace* online.

## Author’s contributions

G.F.R., B.C., M.P., and G.Y.H.L. conceived and designed the analysis; G.F.R. and B.C. analysed data and drafted the manuscript; and D.A.M., F.F., A.B., G.B., T.B., B.O., T.-F.C., M.V.H., M.P., and G.Y.H.L. revised the manuscript and gave relevant intellectual contribution. All authors read and approved the final manuscript.

## Supplementary Material

euae021_Supplementary_Data

## Data Availability

Data supporting this study by the data contributors Boehringer Ingelheim were made and are available through Vivli, Inc. Access was provided after a proposal was approved by an independent review committee identified for this purpose and after receipt of a signed data sharing agreement.
